# Development and Evaluation of a Framework for Authentic Online Co‐Design: Partnership‐Focussed Principles‐Driven Online Co‐Design

**DOI:** 10.1111/hex.14138

**Published:** 2024-07-09

**Authors:** Free Coulston, Alicia Spittle, Cassie McDonald, Rachel Toovey, Kate L. Cameron, Kimberley Attard, Loni Binstock, Isaac Fletcher, Adie Delaney, Tayla Murphy, Caroline Keating, Kath Sellick

**Affiliations:** ^1^ Faculty of Medicine, Dentistry and Health Sciences The University of Melbourne Parkville Victoria Australia; ^2^ Clinical Sciences Murdoch Children's Research Institute Parkville Victoria Australia; ^3^ Newborn Services The Royal Women's Hospital Parkville Victoria Australia; ^4^ Allied Health Alfred Health Melbourne Victoria Australia; ^5^ Consumer Advisory Group, Centre for Research Excellence in Newborn Medicine Murdoch Children's Research Institute Parkville Victoria Australia

**Keywords:** co‐design, end‐users, mixed‐methods, online, patient and public involvement and engagement

## Abstract

**Introduction:**

Co‐design in health research involves patient and public involvement and engagement (PPIE) in intervention or service design. Traditionally, co‐design is undertaken in‐person; however, exploring online delivery is warranted. PPIE in co‐design must be considered carefully, and assumptions that in‐person approaches will transition automatically to an online environment should be avoided. Currently, there are a lack of evidence‐informed approaches to facilitating co‐design online. This study aimed to develop and evaluate a framework for authentically adapting health research co‐design into an online environment.

**Materials and Methods:**

The initial framework was developed through a literature review, synthesis of in‐person co‐design principles, and alignment of online strategies. The framework was then applied to a co‐design project with 10 participants across relevant PPIE groups (end‐users [*n* = 4], clinicians [*n* = 2], coaches [*n* = 2] and clinician−researchers [*n* = 2]). Participants' experiences of the online co‐design process were evaluated via a mixed‐methods design using surveys and semi‐structured interviews. Evaluation data were analysed using descriptive statistics and reflexive thematic analysis to inform a revised framework.

**Results:**

The developed framework, Partnership‐focussed Principles‐driven Online co‐Design (P‐POD) was used to design eight 90 min online co‐design workshops. Evaluation data involved 46 survey responses, and eight participants were interviewed on project completion. Survey data indicated that the process was satisfying, engaging and adhered to the P‐POD framework. Themes derived from interview data describe a respectful and collaborative online culture, valuing of diverse perspectives and space for healthy debate, how power was perceived as being shared but not equal and multiple definitions of success within and beyond the process. A final, refined P‐POD framework is presented.

**Conclusion:**

With evaluation of the initial P‐POD framework showing evidence of adherence to co‐design principles, positive participant experiences and goal achievement for both the project and the participants, the refined P‐POD framework may be used and evaluated within future intervention or service design.

**Patient or Public Contribution:**

This study involved the participants (end‐users, clinicians and service providers) in the co‐design process described, interpretation of the results through member‐checking interview responses, assisting in development of the final framework and as co‐authors for this manuscript.

## 1 Introduction

Patient and public involvement and engagement (PPIE) in health research may engage different groups, including the population that is the intended target of the intervention, service providers, academics, funders and policy makers [1, 2, 3, 4, 5]. PPIE in health research and intervention design is advocated for two reasons. First, it enables people most affected by the research to provide their perspective on constructs such as barriers, facilitators and outcomes [4, 6]. This increases the likelihood that the intervention will be relevant and acceptable, thus reducing the knowledge‐to‐practice gap and improving uptake of healthcare initiatives [1, 6, 7, 8, 9, 10, 11, 12, 13]. Second, meaningful involvement of end‐users in the research that affects them is as an ethical imperative, as evidenced in the disability rights call to action ‘nothing about us without us’ [11, 14, 15, 16, 17, 18]. However, PPIE must be considered carefully. Tokenistic or ill‐defined approaches can create distrust and may further perpetuate problematic power distributions such as research *on* end‐users, rather than *with* end‐users [16, 19, 20].

Co‐design, a process that brings people together to develop shared solutions to identified challenges, can facilitate PPIE in health research [21, 22]. Co‐design involves partnering with end‐users and other groups to create solutions that meet their identified needs. Furthermore, co‐design centres end‐users as true collaborators who drive the design and decision‐making, ensuring that their needs and experiences are at the centre of the process [9, 11, 23, 24].

Co‐design in health research is often operationalised into three distinct, yet often iterative, phases: (1) an information gathering phase to understand the problem, including the needs of the end‐users, (2) a series of workshops in collaboration with end‐users and other groups to create and test potential solutions resulting in a final intervention design and (3) evaluation of the co‐design process and its outcomes [13, 22, 25, 26].

Traditionally, the Phase 2 workshops of co‐design processes are undertaken in‐person. However, in recent years, co‐design has moved to an online environment, particularly during the COVID‐19 pandemic. Exploring online methods is vital to capitalise on the convenience, accessibility and creative use of technology that online approaches offer and to ensure that PPIE continues to enrich health research regardless of restrictions on face‐to‐face attendance [27, 28]. However, there is limited methodological literature describing how to authentically transition co‐design workshops into an online environment, both to ensure that the people involved have positive experiences engaging in an online environment and that relevant, high‐quality outcomes are produced.

This study describes the development of a framework for Phase 2 co‐design workshops delivered in an online environment. Evaluation (Phase 3) of this novel framework is essential to understand if it retains the authenticity and benefits of traditional in‐person approaches [29]. The information‐gathering Phase 1 data informing the Phase 2 workshops is described separately in another paper [30].

The primary objectives of this study are to:

1. Describe the development of the Partnership‐focussed Principles‐driven Online co‐Design (P‐POD) framework,
2. Evaluate P‐POD to assess its alignment with the guiding principles of traditional in‐person co‐design processes and to understand the participants' experience, and
3. Propose a final P‐POD framework for future use and evaluation.

## 2 Materials and Methods

This research sits within a community‐based participatory research (CBPR) approach [2]. CBPR aims to foster partnerships between researchers and other groups (particularly end‐users) to co‐create and translate knowledge into practice [2]. The current study was conducted in the context of developing a community circus training programme for children, families and coaches to optimise outcomes for children born preterm. Prior publications describing the information gathering phase of this study are available [30, 31]. This study received ethical approval from The Royal Children's Hospital Human Research and Ethics Committee (Ethics approval number: HREC/15/RCHM/110).

To develop and evaluate the P‐POD framework, four key steps were undertaken:

- Step 1: Synthesis of relevant literature.
- Step 2: Creation of an initial P‐POD framework.
- Step 3: Use of the initial P‐POD framework.
- Step 4: Evaluation of the initial P‐POD framework.

### 2.1 Step 1: Synthesis of Relevant Literature

The aim was to understand the guiding principles underpinning in‐person co‐design processes and to identify strategies used to uphold these principles throughout the co‐design workshops. A modified scoping review approach [32] was taken, whereby co‐design literature was searched in health‐related databases (Medline [OVID], Cinahl [Ebsco] and Scopus). Inclusion criteria included peer‐reviewed or grey literature describing co‐design methods. Extracted data included principles and strategies that could be used within workshops to inform engagement, decision‐making and design activities.

### 2.2 Step 2: Creation of an Initial P‐POD Framework

Extracted data were then grouped into categories, and each category was synthesised into a final ‘guiding principle’. Strategies amenable to an online environment from the literature and from the research team's experience were also categorised with each guiding principle to develop the initial P‐POD framework (detailed in Section Results, 3 and Appendix SA).

### 2.3 Step 3: Use of the Initial P‐POD Framework

The P‐POD framework was then utilised to plan a series of research co‐design workshops. Agendas for the first two co‐design workshops were planned by the research team, while the remaining agendas were developed by the participants within the co‐design workshops using the P‐POD principles (Appendix S, B).

Participants for the co‐design workshops were purposively selected from the prior information gathering study [30] using maximum‐variation sampling based on survey and interview responses. As per the P‐POD framework, in sampling, the aim was to consider who might be most affected by the intervention, as well as having diversity in PPIE groups to explore important perspectives and types of expertise [3, 16]. Participants selected are described in detail in Section Results, 3.

The overall sample size (*n* = 10) was based on recommendations for traditional co‐design practices [3] and on the practicalities of ‘seeing’ everyone on a single screen during workshops. We chose to have higher numbers of end‐users to recognise and address potential power imbalances that could occur within the group. Informed consent to participate in the project was sought in writing and reconfirmed verbally. End‐users and service providers were reimbursed AUD$50 per workshop attended, while the academics were involved as part of their university roles.

### 2.4 Step 4: Evaluation of the P‐POD Framework

**Figure 1:**
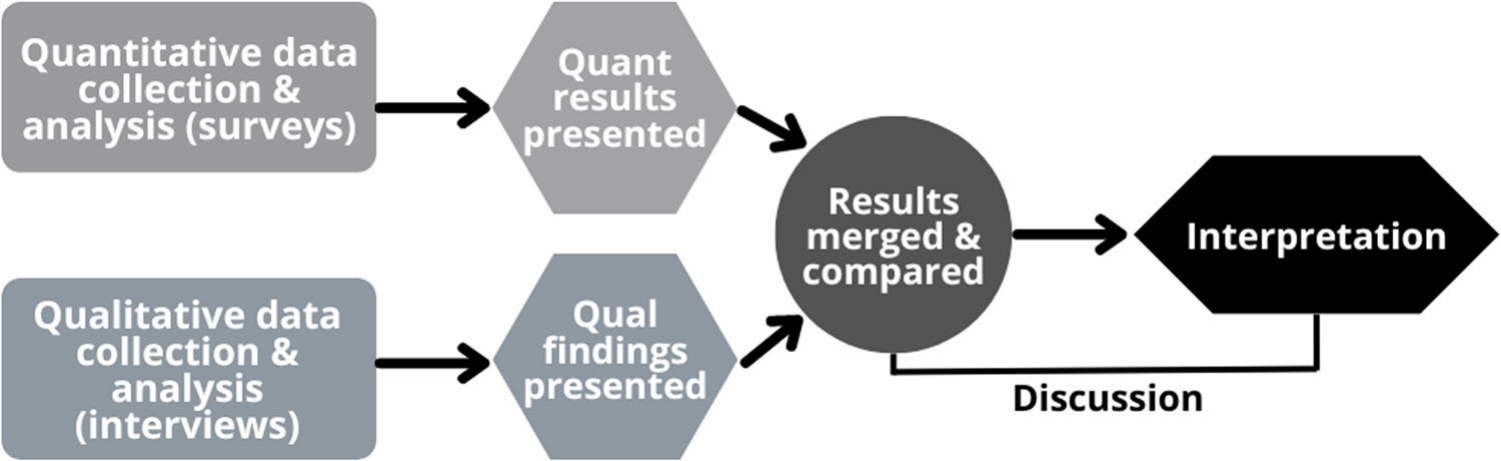
Convergent mixed‐methods design employed in the evaluation of the online co‐design process.

To evaluate participants' experiences of the online co‐design process, a convergent mixed‐methods design using surveys and semi‐structured interviews was utilised (Figure 1). This evaluation method allowed experts to provide support to the lead author in each arm of the study [33].

Survey and interview guides (Appendix SC) were developed based on the literature on evaluating co‐design [3, 8, 11, 13, 22, 25, 34, 35]. Anonymous surveys completed each workshop via REDCap (Research Electronic Data Capture, version 12.5.16 hosted at Murdoch Children's Research Institute, Melbourne, Australia) [36, 37], evaluated workshop adherence to the P‐POD framework, participant satisfaction and suggestions for improvement. Descriptive statistics were performed (Microsoft Excel for Mac, 2023 version 16.71) by F.C. under the guidance of a statistician.

Semi‐structured interviews were conducted after completion of the workshops to explore participants' experiences of the online co‐design process. A phenomenological approach [38] informed the development of the interview questions, including positive aspects and challenges, goal achievement and perceived learning and impact (Appendix SC). A research team member experienced in qualitative research and interviewing but not involved with the co‐design process (K.C.) conducted the interviews via Zoom between September and December 2021. Audio recordings were captured via Zoom and transcribed verbatim by OutScribe (an external transcription company). All identifying information except for the PPIE group was removed before analysis. Analysis was undertaken using the six phases of reflexive thematic analysis as described by Braun and Clarke [39]. A detailed description of the analysis process, including reflexive statements by the analysts F.C., C.M. and K.C., is provided in Appendix SD. Member checking involved interviewees reviewing the developed themes and all co‐design team members reviewing the final P‐POD framework. All participants reported that the themes and final framework resonated with their experiences, and no changes were requested.

The analysed quantitative survey data and qualitative interview data were then bought together at the interpretation stage in the discussion, facilitating a depth of understanding that aligns with the recommendations in the literature for the use of mixed‐methods in evaluation of co‐design and PPIE processes [16, 23, 33].

## 3 Results

### 3.1 Synthesis of Relevant Literature and Creation of the Initial P‐POD Framework

**Figure 2:**
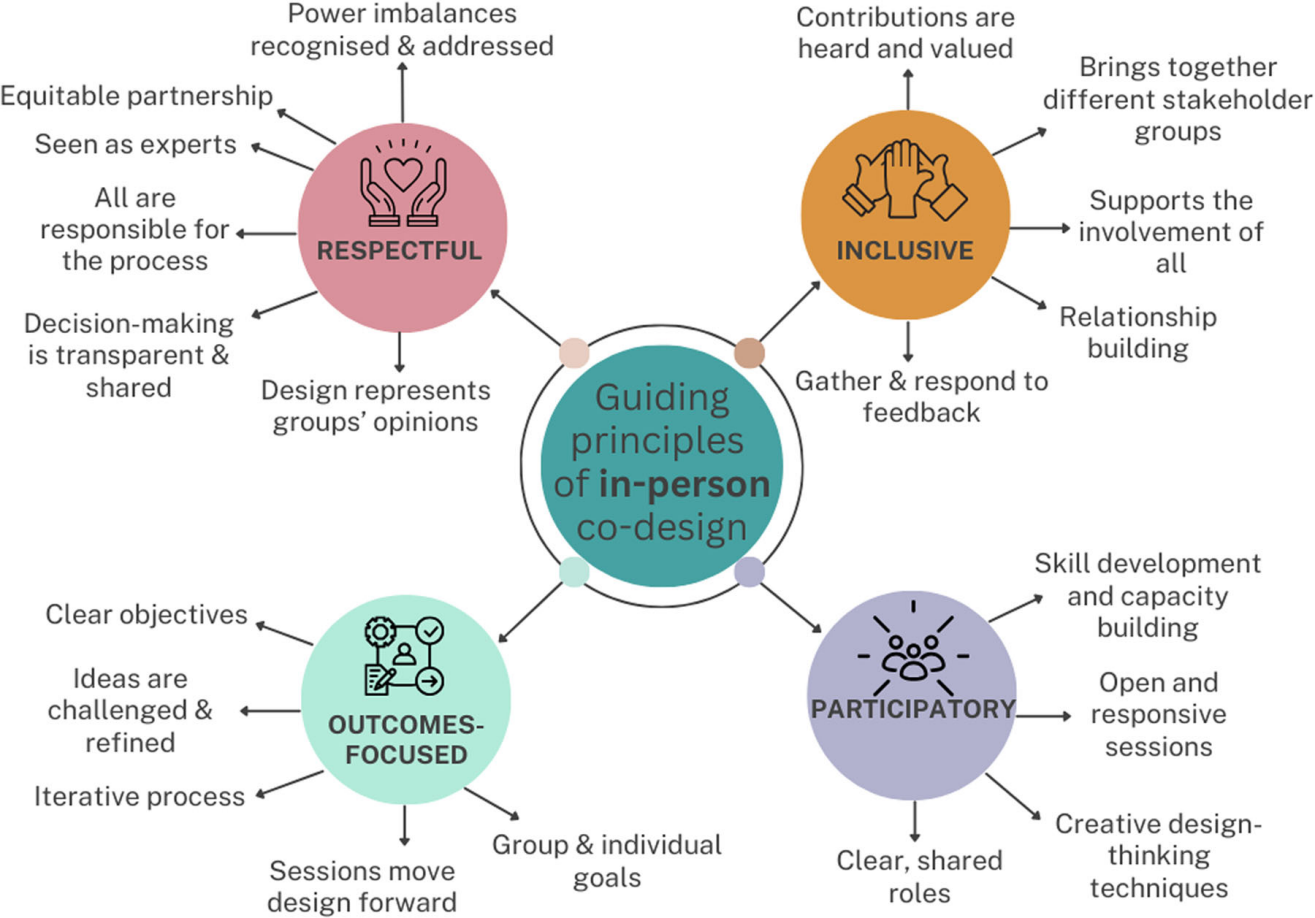
The guiding principles for the initial P‐POD framework.

Synthesis of the literature resulted in four final guiding principles for P‐POD: (1) being respectful [3, 11, 16, 18, 22, 23, 29, 40, 41], (2) being inclusive [1, 11, 18, 22, 29, 41, 42], (3) being participatory [1, 3, 11, 18, 22, 23, 29, 40, 42] and (4) being outcome‐focused [1, 3, 11, 16, 22, 29, 40] (Figure 2). Further detail on the associated strategies for each principle can be found in Appendix SA.

### 3.2 Use of the Initial P‐POD Framework

Eight 90‐min online co‐design workshops, developed using the P‐POD framework (Figure 2, Appendix SA), were conducted from June to September 2021. Initially, five workshops were planned; however, in the fifth workshop, seven participants voted to continue for another three workshops to finalise key intervention details. The resulting co‐designed intervention can be viewed in Appendix SE.

Participants in the co‐design team included:

- Four end‐users who brought their experience of parenting an extremely preterm child and their experience of accessing and participating in recreational physical activities with their preschool‐aged children in Victoria, Australia.
- Two coaches who brought their service‐provision experience of teaching circus to preschool‐aged children and of managing preschool circus programmes in two diverse locations in Australia; one a small independent company in a rural town and the other, one of the largest circus schools in Australia, located in a major capital city. Furthermore, one coach had a background in trauma‐informed education, and the other had training in occupational therapy.
- Two clinicians, one who was a paediatric psychologist working in a hospital setting and a not‐for‐profit Early Childhood Approach National Disability Insurance Scheme setting, and the other, a physiotherapist with a special interest in paediatrics working in a private practice setting.
- Two academics, both researchers and physiotherapists, with backgrounds in teaching recreational physical activity to young children (circus: F.C. and dance: C.M.), and experience in qualitative and mixed‐methods research. F.C. was the project lead and workshop facilitator, and C.M. had prior experience in co‐design and provided facilitation support during workshops.

### 3.3 Evaluation of the P‐POD Framework

Survey and interview response rates are described in Table 1. Interviews had a mean duration of 37 min (range 20−50 min). One academic (F.C.) did not contribute data to the evaluation process due to their role as project lead.

**Table 1:** Evaluation data were collected from participants.

Co‐design team members	End‐users (*n* = 4)	Service providers (*n* = 4)	Academics (*n* = 2)	Not identified^a^
Survey responses (*n* = 46)	18	19	7	2
Interviews (*n* = 7)	3	3	1	

^a^ Team members who did not indicate their PPIE group in the survey responses.

**Figure 3:**
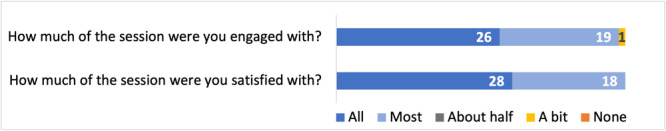
Anonymous survey responses related to participant engagement and satisfaction with the online co‐design workshops.

**Figure 4:**
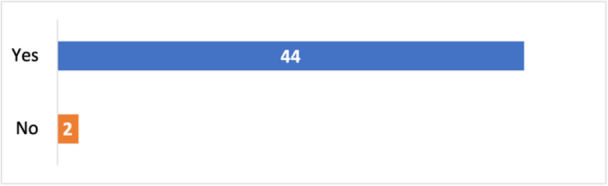
Anonymous survey responses to the question ‘Overall, do you feel you are gaining new skills or knowledge that is useful?’

Satisfaction and engagement with the workshops were high (Figure 3) and indicated participants felt they were gaining new skills and knowledge (Figure 4).

**Figure 5:**
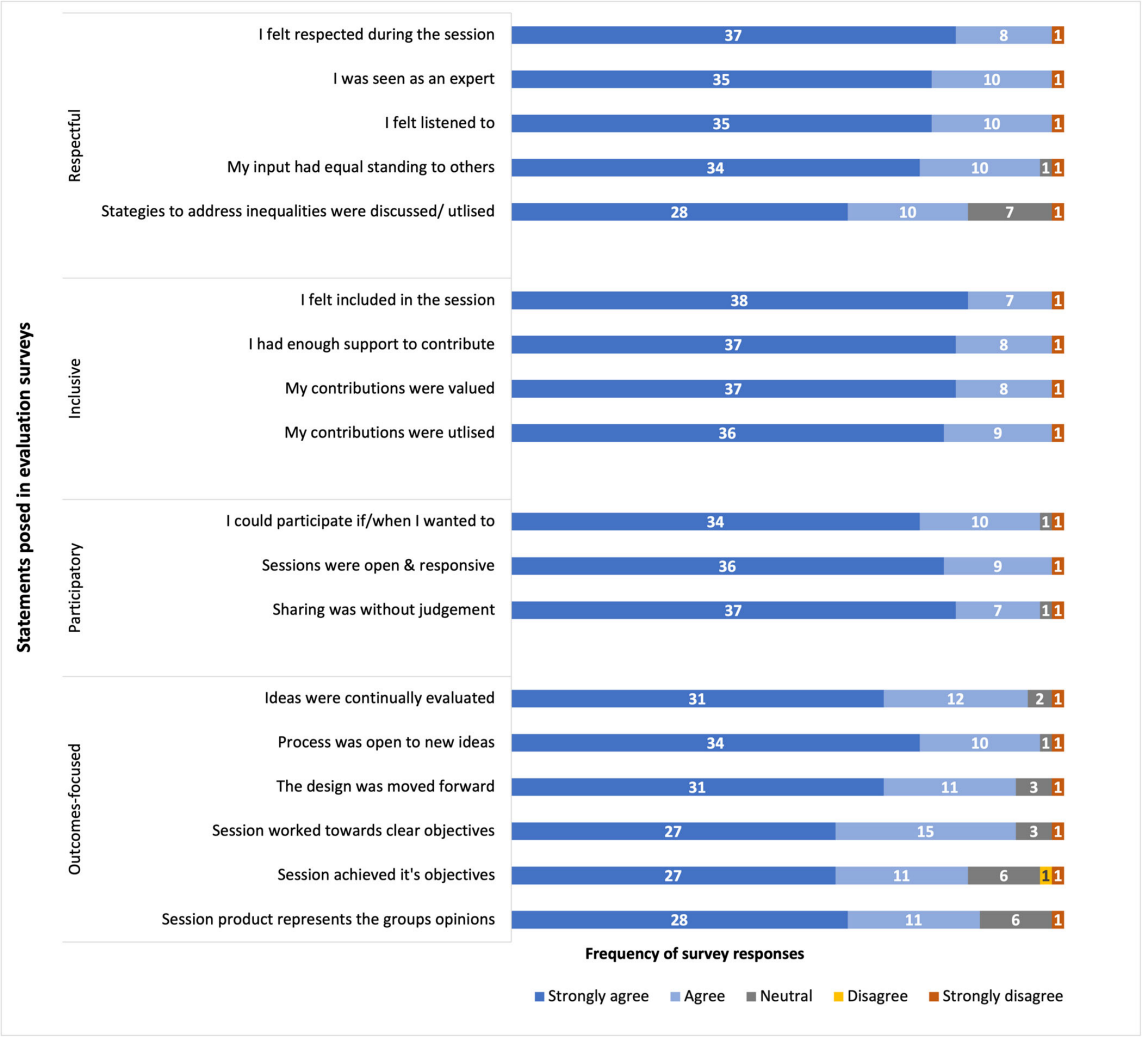
Anonymous survey responses related to the adherence of online co‐design workshops to guiding principles.

More than 80% of responses related to the workshops' alignment with the P‐POD framework indicated ‘agree’ or ‘strongly agree’ (Figure 5).

Four themes were developed from the interview data which explored participants' experiences of the online co‐design workshops.

#### 3.3.1 Theme 1. Co‐Curation of the Online Culture: Setting the Scene for Collaboration

An online environment was viewed as convenient and enabling a nationwide team to participate. The culture within this online environment was described as genuinely collaborative, inclusive, respectful and productive. Participants' reflections on particular elements that curated this culture included ‘scene setting’ through the work done before commencing the process and the initial workshops and the development of relationships between participants.

At the start it was all like strangers or tiptoeing, and then as it got in, we all sort of became like a group, just like a friendship group, catching up every week and continuing as a team with a shared goal.

(ParentB)

##### 3.3.1.1 Scene Setting in Online Co‐Design

A prior information gathering study [30] was credited as resulting in a clear vision and goal for the co‐design process as well as providing a rich data set to inform the intervention design. As all workshop participants were sampled from this study, they felt this resulted in an invested and enthusiastic co‐design team with the required expertise (both professional and lived experience), who were familiar with the project.

Many participants reported that the initial workshops were instrumental in curating the online culture and that the emphasis on lived and professional experiences being of equal importance contributed to an environment of respect and enabled early understanding of the rich backgrounds of each team member.

Getting an idea of everyone's backgrounds in the first session, ‘hey this is me, this is a little bit about me’, so you can sort of understand where everyone's points of views are coming from was definitely helpful. Because it was a really big mix of backgrounds … So I think having an understanding of people's backgrounds as well as why they're involved in the program, that was also helpful.

(ClinicianB)

Participants described how the expectations of the process were made explicit in these early stages facilitating a clear, unified goal for the co‐design. For example, guidelines to govern group conduct were collaboratively constructed by the group through an exercise using online collaborative software (Padlet: a type of productivity software and virtual whiteboard described in Appendix SA [43]). Many participants also commented on the upskilling in co‐design theory, which introduced the expectation early of active participation, and strongly influenced the online environment and culture moving forward.

##### 3.3.1.2 Facilitating Relationship Building in Online Co‐Design

The use of breakout rooms via the online teleconferencing platform (Zoom) throughout the process was instrumental in developing relationships. Participants also identified that familiarity with some team members from the information gathering study [30] helped to accelerate relationship building. Parents in particular spoke of their appreciation of having other parents in the team and how the shared experiences voiced within the process were felt to validate their own experiences and input.

Some participants felt that a hybrid delivery may have enabled a more natural conversational manner and potentially enhanced relationship building. However, they emphasised that retaining the option for online attendance was essential to enable access for all team members. Additionally, some participants reflected that a key benefit of the online approach was that participants could have breaks when needed without drawing attention to themselves and felt that this was key to their participation in the process.

#### 3.3.2 Theme 2. Difference Without Discord: Valuing Diverse Perspectives and ‘Making Room for Healthy Debate’

This theme describes how participants valued the diversity within the group and how their contributions enriched and challenged each other during the workshops.

The team that was put together was very supportive and comfortable and interested and engaged. Everyone was very open to sharing experiences and knowledge, and very respectful of knowledge as well.

(CoachA)

##### 3.3.2.1 Valuing Diverse Perspectives in Online Co‐Design

All participants described valuing the team's diverse backgrounds and how this impacted on their experience. The wealth of both lived and professional experiences opened up new ideas and perspectives that enriched the intervention design and, for some, relieved feelings of anxiety about the complexity of the process and the breadth of expertise required to achieve the project's goal.

Many participants specifically mentioned the parents' contributions and perspectives as being invaluable, as their insights into potential solutions based on their experiences were honest and thoughtful. However, a couple of participants highlighted that a potential risk in this process was that the resulting intervention design may be limited to the groups' own experiences and prompted thoughts around the generalisability of the resulting design.

##### 3.3.2.2 Working With Diverse Perspectives in Online Co‐Design

All participants spoke positively of the diverse perspectives and opinions shared and of how this was managed in the online environment. Participants appreciated the ‘room for healthy debate’ and described how challenges to ideas and assumptions were positive and meaningful enough to prompt them to reconsider their stance.

I had an idea of what I wanted, but once we batted it round the group, I was like, ‘Yeah, no, I'm wrong. We should do it this way. That's going to be way better.’ Repetitively. And I think that happened to pretty much everyone at some stage. I don't see that happening as much in traditional groups as it happened in this one. It was cool.

(CoachA)

Tools that participants felt enabled this healthy debate while attributing clear value to each person's perspectives were the Consensus Decision‐Making framework [44], the clarity of the project's overall goal and the role of workshop facilitator. These were felt to support respectful collaboration and move the co‐design forward, even when aspects of the design needed time for debate. Participants also commented that the final intervention was able to cater for multiple perspectives without significant compromise.

I liked the process in that it never really came to a head, it was just a process of continuing to discuss our viewpoints and then coming to [consensus] … ultimately, we weren't forced to choose a way forward.

(ClinicianA)

#### 3.3.3 Theme 3. ‘Everyone is Equal’ but … Experiences of Power and Agency

This theme describes participants' reflections on the power dynamic within the online co‐design process.

##### 3.3.3.1 A Different Approach to Power in Online Co‐Design

Many participants described their surprise at the power dynamic within the co‐design workshops and how it was unlike other experiences of participating in meetings and research processes. They commented on their surprise that the facilitator stood back from a traditional leadership role and instead handed over the direction of the design and decision‐making to the group.

It wasn't like, I'll give my opinion but you're all going to make whatever decision you want […] this was a genuine respect and inclusion of everybody's thoughts and opinions that was going to drive the outcome of the project, which again, just blew me away that that could be successful. And that that could work.

(ParentA)

For the one participant who had participated in other co‐design processes, this was still novel, as in their experience, in times of key decision‐making the project lead would step in with a final decision. Participants felt they had power to direct the design but also agency regarding their individual contributions to the process. Being able to choose when and how their experience and perspectives were shared was important, as was being welcome and encouraged to contribute but not compelled. The variety of methods to contribute in the online environment were noted by many participants as key to their involvement and a strength of the process. Particularly, the online anonymous voting, small group discussions and activities via breakout rooms in the online platform were noted by participants as ensuring the quieter voices were heard and fed back into the process.

You feel like if you had something to say you could say it, it wasn't like dominated by anyone and Free was really good at sharing that around, making sure everyone had their say. And even opening it up if you don't want to say it on the screen, email her or if you don't want to, you could put it in that question box at the end of each session of what you feel. […] We did a lot of polls anonymously which was really good too just to see where the group's at and you didn't feel like you were judged if you had a different opinion, not that we really had that vibe anyway.

(ParentB)

##### 3.3.3.2 Leadership in Online Co‐Design

Even though participants reported agency in contributions, design discussions and decision‐making, there was a clear power difference between the co‐design participants and F.C.'s roles of project lead and workshop facilitator. As project lead, having PhD requirements to set the research question and obtain ethical approval before beginning the study, power was retained by F.C. As a result, some participants described lacking a sense of how the process would all come together and expressed that having a clearer week‐to‐week ‘map’ would have been helpful.

The role of the workshop facilitator (including administration tasks between workshops) was commented on by many participants as being essential for enabling meaningful contributions, progressing forward in the design process, and valuing the time of the team members. Although F.C. took on this role intially, offers were made throughout the remaining workshops for other participants to step into parts of this role in an attempt to address power imbalances. Participants commented on appreciating these attempts but being unsure how successful this role sharing was in practice. Although many participants did contribute by adding to the collaborative software, writing agenda items and reading minutes, some participants reflected that taking on additional administration tasks was not possible as they were already at capacity with their current contributions.

If the reason was that they felt that they had genuine choice and didn't want to, that's great. But if there were other barriers, like someone didn't feel confident or they wanted more support or training to step into the role […] maybe we didn't quite get that part of it right, that we were offering opportunity but not giving people enough support to really take it up.

(AcademicB)

However, a key result of these attempts was that participants described the action of creating opportunities for role sharing allowed ‘the rest of us to step forward a bit more than we would have, I think’ (CoachA).

#### 3.3.4 Theme 4. ‘It was Quite Enlightening’: Success is More Than the Co‐Designed Product

This theme describes how participants defined and experienced success in the online co‐design process.

I think it definitely helped me grow as a person definitely, which is kind of weird to say in like eight sessions. But yeah, it was quite enlightening […] it's opening my world to different things.

(ParentB)

##### 3.3.4.1 Goal Attainment in Online Co‐Design

All participants felt that the process was successful in its goal of designing an intervention to increase physical activity participation for preschool‐aged children born preterm. Some participants described wanting the process to continue for longer but also described a tension between committing further time, and their actual capacity to do so, and reflected on whether more time would have necessarily resulted in a high‐quality outcome. Participants often described a sense of ownership of the design output and that they felt seeing the design in action (e.g., testing the co‐designed intervention in a real‐world setting) was a critical part of being able to definitively say the project was a success.

Participants also recorded individual goals on the collaborative software early in the process. End‐users spoke strongly of perceived success in their personal goals of reciprocity to the medical community—wanting to ‘give back’ in return from having benefitted from the results of prior research. One parent described how they had also developed confidence in their end‐user expertise through participating in the co‐design.

You kind of just do what you do as a parent and you don't realise that it might be helpful to share that with other people. So this co‐design process gave me some confidence there […] that's what this co‐design process made me see is that, what we do can be quite valuable and that it might be helpful to other families.

(ParentA)

Both clinicians also reflected that the process had reaffirmed for them the importance ‘of [understanding] what's going on for parents, what their perspective is and how important that is to inclusion’ (ClinicianA) and how programme design can be modified to ameliorate barriers for parents.

##### 3.3.4.2 Personal and Professional Impacts of Online Co‐Design

All participants reported impact and benefits beyond the workshops, describing an appreciation of gaining transferable skills and knowledge that they were intending to integrate into their professional and/or personal lives. Many participants reflected on their learnings of co‐design as being a strong takeaway, and some described how they would integrate online co‐design into their future professional lives.

I'm a project manager myself, so just having another view of how you can work with stakeholders and engage people in a really collaborative way that minimises that power imbalance between possible relationships, is always a good, practical experience for me.

(CoachA)

Many participants spoke of utilising the Consensus Decision‐Making framework and its value in regard to honouring diverse perspectives but also as disrupting the traditional power structures in key decision‐making processes.

I think that whole concept of decision‐making in a very different way has filtered through for me. I've been trying to be more collaborative in the way I make decisions with my older children, one's a tween and one's a teenager. At work, I've been really trying to back off from being the decision‐maker and collaborate with everybody in a different way. The validation and the respect of other people's opinions is something that you don't see very often.

(ParentA)

**Figure 6:**
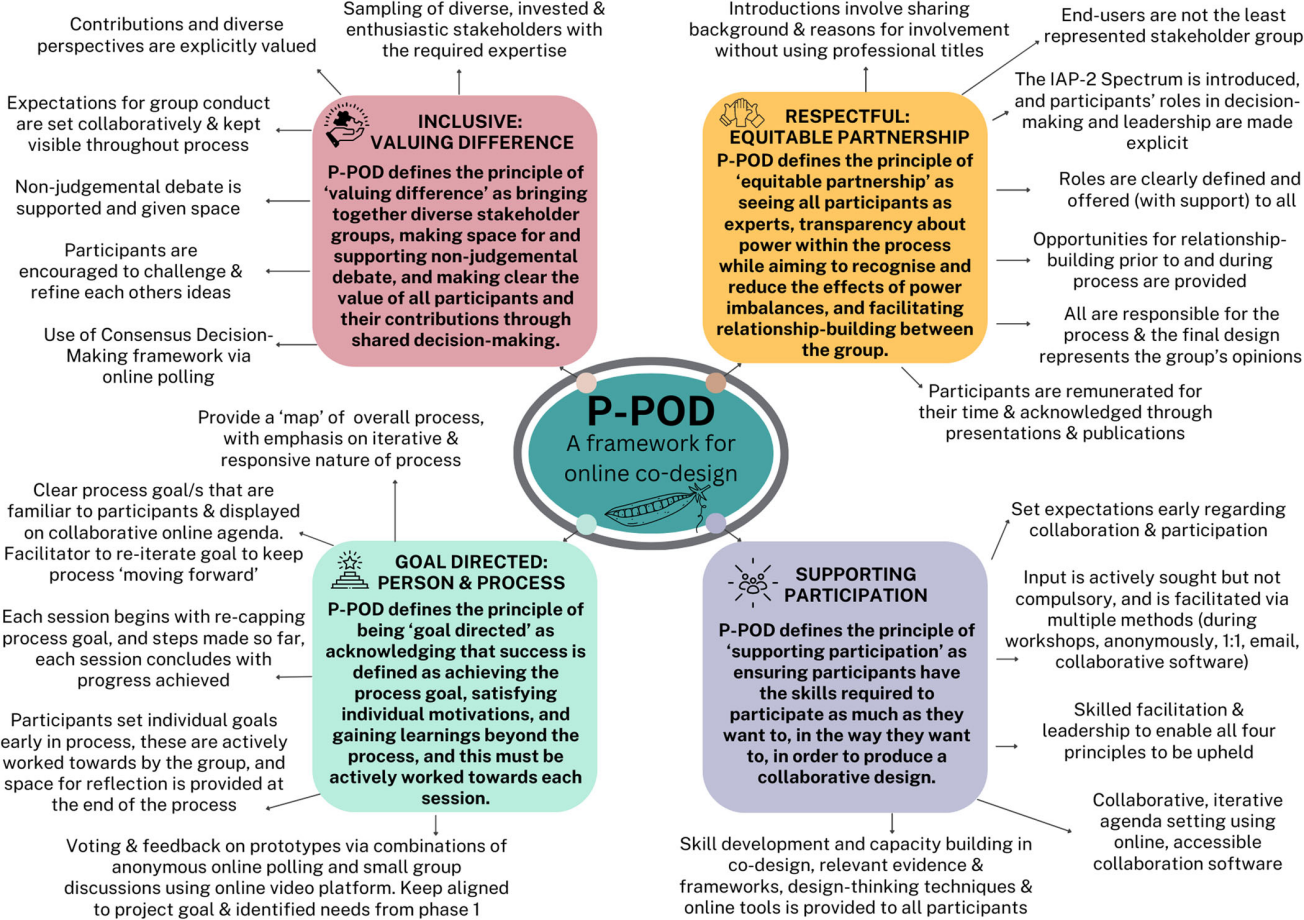
The final Partnership‐focussed Principles‐driven Online co‐Design framework.

Based on these results, the final P‐POD framework for an authentic approach to online co‐design is presented in Figure 6 (associated strategies are detailed in Appendix SF).

## 4 Discussion

This study developed and evaluated a novel framework for online co‐design (P‐POD). The evaluation found that the initial P‐POD framework aligns with principles of in‐person co‐design processes and is satisfying and engaging for participants. Participants experienced the process as having a respectful and collaborative online culture, valuing diverse perspectives and space for healthy debate, power being shared but not equal and multiple definitions of success within and beyond the process. Suggestions for improvement to the initial framework were incorporated into a final P‐POD framework (Figure 6; Appendix SF).

P‐POD's success in ‘being respectful’ is reflected in both the qualitative and quantitative findings. Not only did participants describe the curation of, and participation in, a respectful environment as key to their experience but also they indicated agreement in more than 97% of responses to both the survey statement of ‘feeling respected’ and being ‘seen as an expert’ during online co‐design workshops. Furthermore, one participant described new‐found confidence in their end‐user expertise, which aligns with benefits reported during in‐person co‐design processes [11, 16, 45].

A key element of being respectful is an equitable partnership that is transparent about power distribution and seeks to recognise and reduce the effects of power imbalances. Many P‐POD participants spoke strongly of their leadership in decision‐making and ownership over the design. Participants appreciated attempts to power share through task and role sharing; however, many recognised that more responsibility equated to more burden and expressed that additional responsibility was beyond their capacity to invest. Although participants felt ownership and desire to see the design progress into the testing stage, many participants felt that ultimately, responsibility for the process fell to the project lead. These findings indicate that to facilitate P‐POD some team members (such as the project lead) must assume increased responsibility and complete additional tasks. This prompted the authorship team to discuss and debate: should research teams strive for equality for all members throughout an online research co‐design process? Based on the evaluation findings, the authors posit that power sharing in P‐POD should instead focus on equity and inclusion, rather than equality. This aligns with comments by Farr [46] that power in co‐design should be considered carefully, with the goal being that all participants should benefit from its use, rather than subscribing to an ‘ideal’ of equality. ‘Collective power’ (p. 628) derived from working *with* PPIE groups should result in collaboration, respect and connecting with participants' values [46]. Participants reported these benefits in describing their experiences in P‐POD and therefore moving into the ‘empower’ domain of the IAP2 Spectrum where end‐users who completely drive the research may not always be the ideal, particularly when working with ‘time‐poor’ parents and clinicians.

Participants in P‐POD reported the initial workshops as key to forming the respectful and participatory environment of P‐POD (Theme 1). Pallesen et al. [13] also reflected on the co‐design environment and power sharing as key themes arising from evaluating their in‐person co‐design process. Pallesen et al. reported that participants experienced a sharing of power via collaborative leadership which was actively facilitated by participants influencing the agenda and workshop direction. However, participants in Pallesen et al.'s study reported feelings of anxiety and uncertainty in the first few workshops [13]. In contrast, the positive participant experiences of the initial P‐POD workshops may be due to the familiarity of both the the project and the team members from their involvement in the prior study. This aligns with Pallesen et al.'s suggestion that the easing of their participants' anxiety and uncertainty over time was due to building relationships and gaining familiarity with the processes. Engaging participants in a prior information gathering study and careful design of initial co‐design sessions may accelerate relationship building and confidence within online co‐design teams. Furthermore, specific tools and strategies in the online environment that facilitated this are likely essential to forming the respectful culture that enabled participants to ‘step forward’ early into shared power and agency.

P‐POD participants highlighted the value of diverse perspectives which enriched both the co‐design process and the final intervention design (Theme 2). Appreciation for differing perspectives and expertise is also highlighted in the literature on in‐person codesign [23, 45, 47]. In contrast to P‐POD's evaluation, Gustavsson and Andersson [47] reported an initial reluctance by clinicians to work with end‐users for fear of criticism. Similarly, Hyett et al. [23] describe participants' challenging each other's professional roles. This was not reflected in P‐POD, where clinicians valued the input of parents in challenging some of their assumptions but were not challenged in their own PPIE group role or expertise. This may be due to the respectful and supportive culture that was actively curated in P‐POD, as well as parents' positive experiences in the healthcare system which formed part of their purpose for participating in the co‐design process. Furthermore, undertaking the co‐design via an online platform rather than an in‐person setting may facilitate a more neutral environment that provides less inherent reminders of traditional power hierarchies.

Multiple modalities for contributing synchronously and asynchronously to an online co‐design process may be important to optimise participation from all PPIE groups and aid the sense of feeling valued. Literature on in‐person co‐design reports that feeling heard and valued and experiencing new and stronger social connections are key benefits for participants [11, 16, 45], which was reflected in P‐POD's evaluation. Palmer et al. [45] describe that feeling heard and valued within a co‐design team contributes to a shared purpose and a sense of ‘collective identity’ (p. 253) which motivates the group to connect past experiences towards creating future solutions. P‐POD participants also reported a sense of shared purpose and appreciated the small group discussions in breakout rooms as facilitating the building of relationships (or collective identity) between the team members. However, some participants wondered if a hybrid model for those who could attend face to face might further cement developing relationships. Fails et al. [27] and Kennedy et al. [28] both point out that hybrid models have the potential to act unfavourably on team dynamics, with those joining virtually feeling like ‘outsiders’ (27, p. 3).

Supporting participation by ensuring that participants can choose to contribute when and how they want to, while making space for and supporting nonjudgemental debate, are key elements of P‐POD. Team members felt they had agency in their participation, and this is supported by 98% of responses agreeing that ‘I could participate if/when I wanted to’. Participants discussed the lack of judgement from other team members during sessions and appreciated the room to debate differing opinions. In contrast, Pallesen et al. described their face‐to‐face co‐design process as having an ‘absence of conflict or disagreement’ (13, p. 363), whereas P‐POD seemed to provide an environment where participants felt safe to share and debate diverse perspectives; working towards consensus was a valuable part of the process. Strategies to support and encourage space for healthy debate within an online environment were setting collaborative ground rules using collaborative software and the use of tools such as the Consensus Decision‐Making framework (using anonymous online voting) and Nominal Group Technique (using the collaborative software's scribing and voting capabilities).

Clear goals for the process and the individual team members that are actively worked towards are essential in online co‐design. In Theme 1, participants reflected on the benefit of understanding the project's vision before starting the co‐design process and how this was helpful to keep progressing towards the project goal during the workshops. Tools and strategies in the online environment that enabled this were using collaborative software to keep goals visible at all times and for agenda setting and prototype drafting, and small group discussions in breakout rooms enabling problem solving through key aspects of the design. For the individual's benefit, obtaining new knowledge and skills in co‐design and the use of online collaborative platforms was also identified by participants as important elements of online co‐design, and it is supported by in‐person co‐design literature [11, 16, 23]. P‐POD participants reported gaining knowledge that they would utilise beyond the project, such as an increased understanding of co‐design, consensus decision‐making and appreciation of parents' lived experience.

By contextualising the evaluation results within contemporary literature on in‐person co‐design approaches, the experiences and benefits reported by P‐POD participants align with those described by in‐person co‐design approaches. This provides emerging evidence that P‐POD is a framework that enables an authentic translation of health research co‐design into an online environment. The final P‐POD framework presented in Figure 6 and Appendix SF incorporates participant suggestions for improvement. This framework may be used to conduct future research co‐design workshops in an online environment and will benefit from further evaluation.

## 5 Strengths and Limitations

This study adds to the limited evidence base evaluating online co‐design approaches and provides clear recommendations for future research co‐design aiming to authentically transition to an online environment. However, there are some potential limitations. Firstly, the specific groups that participated in this co‐design process (i.e., circus coaches, clinicians and parents of children born preterm) may limit the generalisability of the findings. There is also a possibility that the participants involved in evaluation interviews felt pressured to speak positively about their experiences due to the influence of social desirability. The primary strategy used to address this issue was having an interviewer unknown to the participants. One participant commented on appreciating the opportunity to reflect on their experiences ‘knowing that I could say bad things too. But there was no bad things’ (ParentB). A collaborative approach to the qualitative data analysis (with three analysts), as well as member checking themes, was used to address the potential for confirmation bias.

Although this research aimed to ‘collaborate’ and not ‘empower’ on the IAP2 Spectrum [24], in future research, the authors would recommend including PPIE input even earlier (e.g., setting the initial research question). This strategy may further address power imbalances without placing an undue burden on participants. As this current study formed part of F.C.'s doctoral research, the research question and focus needed to be decided before the co‐design team was engaged. This issue was also noted in other co‐design research but was not considered detrimental to the process or outcome [23].

Furthermore, children with lived experience of being born preterm were not included as participants due to the age range of the intended intervention and the assumption that parents would be best placed to inform interventions for this age group (3−5 years). However, future research should consider involving older children or adults with this lived experience to further enrich the intervention design. Lastly, as this paper suggests a framework to guide online co‐design rather than a side‐by‐side comparison of face‐to‐face versus online approaches, the decision regarding which approach to choose is multifactorial and beyond the scope of this paper.

## 6 Conclusion

A framework (P‐POD) for authentically adapting health research co‐design into an online environment was developed and evaluated, and a refined framework was presented. The P‐POD framework enables a respectful, inclusive, participatory and outcomes‐focused online co‐design process and appears to impact the lives of the people involved, in a positive and meaningful way. The reported experiences of participants indicate that the benefits of P‐POD align with those reported in the literature regarding in‐person co‐design approaches. This suggests that the P‐POD framework results in an authentic transition of co‐design into an online environment. This study addresses an identified gap in the literature and provides clear guidance for utilising this framework to capitalise on the increased accessibility that online research co‐design approaches provide while ensuring that the process is not tokenistic.

## Author Contributions

**Free Coulston:** conceptualisation, investigation, funding acquisition, writing–original draft, methodology, validation, visualisation, writing–review and editing, data curation, project administration, formal analysis, software. **Alicia Spittle:** conceptualisation, funding acquisition, methodology, writing–review and editing, supervision, resources. **Cassie McDonald:** investigation, writing–review and editing, visualisation, methodology, validation, formal analysis. **Rachel Toovey:** conceptualisation, methodology, validation, writing–review and editing, supervision, resources. **Kate L. Cameron:** writing–review and editing, validation, formal analysis, project administration. **Kimberley Attard:** investigation, writing–review and editing, visualisation, validation. **Loni Binstock:** visualisation, writing–review and editing, investigation, validation. **Isaac Fletcher:** investigation, visualisation, writing–review and editing, validation. **Adie Delaney:** writing–review and editing, visualisation, investigation, validation. **Tayla Murphy:** investigation, writing–review and editing, visualisation, validation. **Caroline Keating:** validation, visualisation, writing–review and editing, investigation. **Kath Sellick:** conceptualisation, funding acquisition, methodology, validation, writing–review and editing, supervision, project administration, resources.

## Ethics Statement

This study received ethical approval from The Royal Children's Hospital Human Research and Ethics Committee (Ethics Approval Number: HREC/15/RCHM/110).

## Conflicts of Interest

The authors declare no conflicts of interest.

## Supporting information

Supporting information.

Supporting information.

Supporting information.

Supporting information.

Supporting information.

Supporting information.

## Data Availability

The data that support the findings of this study are available on request from the corresponding author. The data are not publicly available due to privacy or ethical restrictions.
