# Supplementary material for: Development and Evaluation of a Framework for Authentic Online Co‐Design: Partnership‐Focussed Principles‐Driven Online Co‐Design
Source: Health Expect. 2024 Jul 9;27(4):e14138. doi: 10.1111/hex.14138 (PMC11233779; doi:10.1111/hex.14138)
Supplement: Supplementary file 1 — Supporting information. [file HEX-27-e14138-s003.docx]

Appendix A: Initial P-POD framework.

*Figure A1: The guiding principles for the initial P-POD framework.*


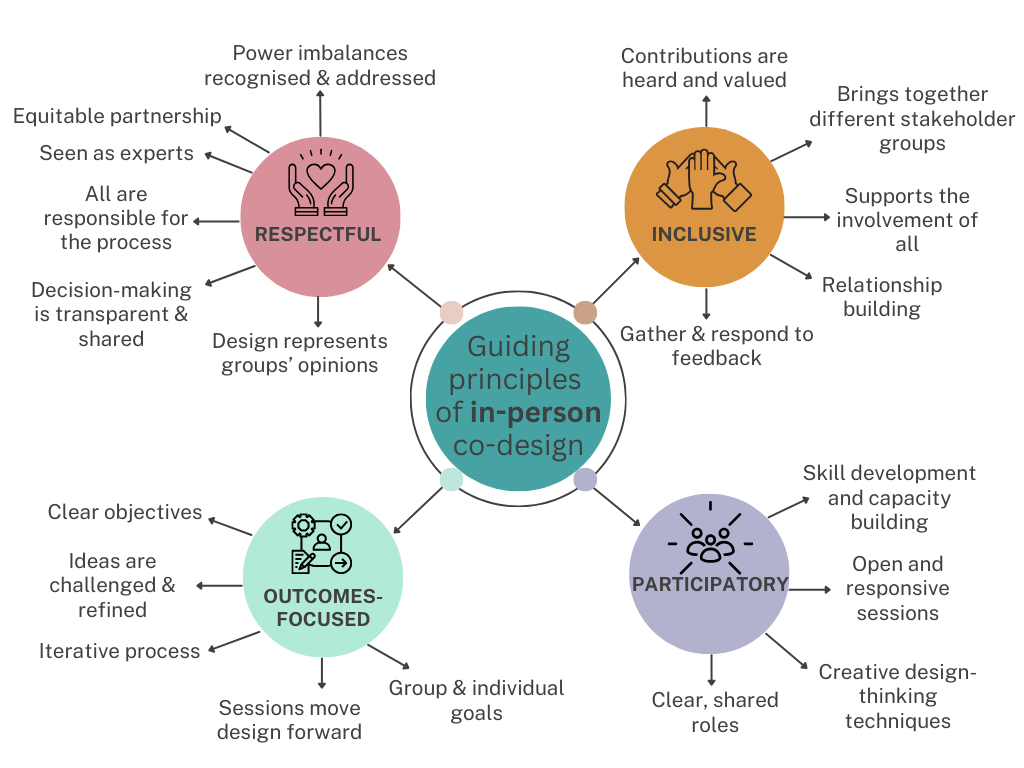


*Table 1: The initial P-POD framework.*

| **Guiding principle & strategies from the literature on in-person approaches.** | | **Strategies selected for an online environment used in the current study.** |
| --- | --- | --- |
| **Being respectful.** | Participants are seen as experts in their lived and/ or professional experience. | Start each online meeting with Acknowledgement of Country^a^ and an acknowledgement of the appreciation and respect for the wealth of knowledge, experience, and skills in the ‘room’. |
|  | Decision making is transparent & shared, design represents groups' opinions. | Consensus Decision Making Framework^1^ used via anonymous videoconferencing platform (Zoom) poll or REDCap^b^ survey. |
|  |  | Use of the TIDieR checklist^c^ to guide design, kept on Padlet^d^ (or other collaborative software) and available to all participants at all times. |
|  | Equitable partnership, potential or perceived power imbalances are recognised and addressed. | Selecting group members so that there is higher number of end-users compared to other stakeholder groups. |
|  |  | During introductions, participants are asked not to include professional titles, but rather to introduce themselves with their relevant lived and/ or professional experience. |
|  |  | Ground rules set collaboratively using collaborative software in the first session. Followed by a discussion on ‘how do we know if these things aren’t happening and how will we negotiate that?’ Rules are kept in the collaborative software and viewed every meeting with time allowed to change/ amend if required. |
|  |  | Explicit promise made in first session by the researchers to 'collaborate' with stakeholders (as per the IAP-2 spectrum): *"We will look to you for advice & innovation, and we will partner in decision-making"*. |
|  |  | Shared roles in sessions; articulating goals and process clearly with stakeholder choice regarding roles. |
|  | All are responsible for the process. | The start of each meeting includes a statement on the shared commitment of participants to the goal of the co-design process. |
| **Being inclusive.** | Bring together different stakeholder groups. | Purposive sampling from different stakeholder groups from the 'Information Gathering Phase'. |
|  |  | Ensure all stakeholders have access to and are upskilled in each online platform/ software used. |
|  | Multiple methods of communication offered. | Contributions can be through:   - group-based online workshops: verbal contributions, written in chat, anonymous in chat, private chat just to facilitators, on collaborative software - outside of group online workshops: part of anonymous feedback survey, emailed to facilitators, 1:1 session (Zoom or phone), on collaborative software, via annonymous surveys. |
|  | Supports the involvement of all participants. | Cameras may be turned off, pseudonyms may be used. |
|  |  | A second facilitator positioned as a support person, both technical but also to clarify information/ activities or for feedback via private chat in teleconferencing platform or via phone call/ text. |
|  |  | Responding to requests for further information/ clarification prior to subsequent sessions. |
|  |  | Time is remunerated. |
|  |  | Offer to include chosen pronouns as part of online name & modelled by academic stakeholders. |
|  | Time is allowed for relationship building. | Breakout rooms in videoconferencing platform utilised often. |
|  |  | Online ice breaker activities.  'Welcome to the Co-design Team' pack sent via mail to team members at the start of the process, including (for example) pen, notepad and chocolate. |
|  | Contributions are heard and valued. | Listening to and considering each idea presented, all ideas noted in collaborative software. |
|  |  | Use of Nominal Group Technique^2^. |
| **Being participatory.** | Skill development & capacity building | Participants upskilled in co-design processes, consensus-building framework & design thinking techniques. |
|  | Open & responsive sessions | Anonymous quality improvement survey embedded into meetings; feedback enacted prior to subsequent sessions. |
|  |  | Collaborative agenda setting via collaborative software. |
|  | Encouraging input through various creative techniques | Use of anonymous polling and up/down-voting ideas via collaborative software to evaluate prototypes. |
|  |  | Ensuring collaborative software accessibility to stakeholders at all times: Padlet was used as agenda and minutes tool, could be accessed by participants at any stage in the process (both during meetings and between). An offer was also made in the first session to send out agendas and minutes via email if preferred. |
|  |  | Utilised design thinking techniques via collaborative software such as idea storms, journey mapping, and add/delete/merge techniques^3^. |
|  | Roles are clearly defined and shared | The scope of the process was clearly outlined during the first meeting, recorded in collaborative software and viewed each meeting. |
|  |  | Shared roles in sessions; articulating goals and process clearly with stakeholder choice regarding roles. |
| **Outcomes focussed** | Clear objectives with attention to group and individual goals | The scope of the process was clearly outlined during the first meeting, the process goal was recorded in the collaborative software and re-articulated at the start of each meeting. |
|  |  | In the first session participants shared what they were hoping to get from the process and these individual goals were recorded in the collaborative software. |
|  | Each session moves the design forward | Each session finished with member checking via reflection on the main findings of the session and how the session had moved the design forward. |
|  | Iterative process with ideas being refined and challenged | Key decisions and latest prototypes recapped at the beginning of each session to direct thinking. |
|  |  | Use of anonymous polling (REDCap or Zoom) and up/down-voting ideas via collaborative software to evaluate prototypes. |

Table notes:

^a^An Acknowledgement of Country is a cultural protocol that is conducted at the beginning of meetings to show respect to Aboriginal and Torres Strait Islander people as the Traditional Owners of Australia^4^.

^b^REDCap (Research Electronic Data Capture hosted at Murdoch Children's Research Institute, Melbourne, Australia) is a secure web platform for managing online databases and surveys^5,6^.

^c^TIDieR (Template for Intervention Description and Replication) is a 12-item checklist devised to improve the completeness of reporting interventions^7^.

^d^Padlet is a type of productivity software and virtual whiteboard that allows collaboration by multiple users and can be password-protected. Key features of Padlet that led to its choice in this project include: shared capacity to add content in real time, easy links to other resources, breakout activities and polling capabilities^8^.

**References**

1. Consensus Decision Making. Seeds for Change. Accessed January 27, 2023. http://www.seedsforchange.org.uk/consensus

2. Harvey N, Holmes CA. Nominal group technique: An effective method for obtaining group consensus. *Int J Nurs Pract*. 2012;18(2):188-194. doi:10.1111/j.1440-172X.2012.02017.x

3. Nesta. Development Impact and You. Development Impact and You. Accessed May 21, 2024. https://diy-toolkit.org/

4. Acknowledgement of Country and Welcome to Country. Reconciliation Australia. Published 2022. Accessed December 15, 2022. https://www.reconciliation.org.au/reconciliation/acknowledgement-of-country-and-welcome-to-country/

5. Harris PA, Taylor R, Minor BL, et al. The REDCap consortium: Building an international community of software platform partners. *J Biomed Inform*. 2019;95:103208. doi:10.1016/j.jbi.2019.103208

6. Harris PA, Taylor R, Thielke R, Payne J, Gonzalez N, Conde JG. Research electronic data capture (REDCap)—A metadata-driven methodology and workflow process for providing translational research informatics support. *J Biomed Inform*. 2009;42(2):377-381. doi:10.1016/j.jbi.2008.08.010

7. Hoffmann TC, Glasziou PP, Boutron I, et al. Better reporting of interventions: template for intervention description and replication (TIDieR) checklist and guide. *BMJ*. 2014;348. doi:10.1136/bmj.g1687

8. Padlet Product. Padlet. Published 2023. Accessed January 27, 2023. https://padlet.com/features
