# Supplementary material for: Development and Evaluation of a Framework for Authentic Online Co‐Design: Partnership‐Focussed Principles‐Driven Online Co‐Design
Source: Health Expect. 2024 Jul 9;27(4):e14138. doi: 10.1111/hex.14138 (PMC11233779; doi:10.1111/hex.14138)
Supplement: Supplementary file 3 — Supporting information. [file HEX-27-e14138-s004.docx]

# Appendix C: Evaluation Survey & Interview Question Guide

## Evaluation Survey

## Evaluation Interview Question Guide (Adapted from questions utilised in the literature^1–7^).

Please note: this is a GUIDE only – this is a semi-structured interview design so please modify as needed to respond to participants.

WELCOME AND INTRODUCTIONS

**Aim:** To make participants feel at ease and ensure they are clear about what to expect and the aims of the interview

- Thank them for giving up their time to speak with us (make them feel welcomed and valued)
- Introductions
  - *My name is [insert], I am assisting with the evaluation of the co-design process you have just been a part of, and I will be asking you some questions about your experiences*
- Provide a very brief description of the research due to pre-existing knowledge of the project & explain how we will use their insights
  - *It’s important to evaluate the process of co-design from the participants’ perspective to understand the benefits and challenges of this method, where improvements can be made, and how it affects those who use it.*
- Explain how the interview will work (format and length):
  - *The interview will take approximately 30-45 minutes.*
  - *I can repeat or clarify questions, if I have been unclear, however you can choose to not answer certain questions, just let me know and I will move on.*
  - *There are no right or wrong or silly answers, I would like you to answer as richly and fully as possible, and I will ask some follow up questions as we go to assist this.*
  - *You can take a break or stop at any time, please just let me know.*
- Re-affirm consent verbally, allow for any questions before asking Interview Questions.
  - *Do you mind if I record this interview so that I can concentrate on what you are saying? PRESS RECORD*
  - *Did you have any further questions before we get started?*
  - *Do you voluntarily consent to continue with the interview?*

**INTERVIEW QUESTIONS**

**Aim:** To gather insights that will help achieve the study aims.

Prompting and probing will be asked based on the participants’ response. Before moving to a new topic, the interviewer will summarise the main points of the participants’ answer to check they are representing their intention correctly.

1. *Now, Free has told me that you have/ haven’t participated in a co-design project before.*
   1. *Have (IF only):*
      1. *Can you describe your understanding of co-design for me?*
      2. *How did The Co-Design Team’s process compare to your previous experience?*
      3. *How do you think an online context impacted the co-design process?*
   2. *Haven’t (all others):*
      1. *Can you describe your understanding of co-design for me?*
      2. *How do you think an online context impacted the co-design process?*
2. *Can you describe your experience of the co-design process for this project, from the initial survey, interview, focus group (parents only) through to the Co-design Team?*

- *Probing questions (if needed)*
  - *How did it make you feel? What is an example of the best part? What about the worst part?*

*Thinking now specifically about The Co-Design Team Meetings:*

1. *How do you feel about what the Co-Design Team achieved in regards to it’s goal:* to co-design an intervention using circus activities that improves physical activity participation for pre-schoolers born preterm, using the information collected from parents, health professionals & circus coaches, and the combined expertise in the Co-design Team.

- *Probing questions (if needed)*
  - *Would you consider the process a success? Can you elaborate a little on that?*

1. *In the first session, you were asked what you were hoping to get out of this process and you described…..*
   - *Participant 1: wanting to learn more about co-design and network with likeminded people*
   - *Participant 2: wanting to ‘give back’ as a result of the care you and your boys received*
   - *Participant 3: interest in research and keen to contribute to the development of a program which is evidence-based and could impact policy*
   - *Participant 4: wanting to create a program which is appropriate and inclusive for kids born preterm and offers parents a chance to connect*
   - *Participant 5: seeing a lot of potential for this project to make a difference in the community*
   - *Participant 6: hoping to create a program that would benefit future clients*
   - *Participant 7: wanting to assist in creating a program that would improve outcomes for children born preterm*
   - *Participant 8: wanting to work with and learn from the team, hoping to implement the circus program we codesign.*
   1. *Can you describe how this may or may not have occurred?*
   2. *What other expectations did you have of the co-design process?*
2. *What do you think worked well about the co-design process?*

- *Probing questions (if needed):*
  - *What was the most satisfying or enjoyable experience? What were some of the advantages to participating in this co-design project? What did aspects did you enjoy/find interesting or useful?*

1. *What do you think did not work so well about the co-design process?*

- *Probing questions (if needed):*
  - *What was the most frustrating or unenjoyable experience? What were the challenges/barriers and what (if any) steps were taken to overcome these challenges?*
  - *Do you think everyone was engaged in the workshops? Do you think this changed over time? If not, what do you think might have prevented people from engaging with the workshops?*
  - *What should we do differently next time we use co-design?*

1. *Has anything changed in the way you live, work or think about things as a result of participating in this project?*

- *Probing questions (if needed):*
  - *Did being involved in the project have any impact on you, whether positive or negative?*
  - *New knowledge, new skills, new networks, new understandings, new opportunities, new possibilities?*

1. *How likely is it that you would participate in another project that uses co-design after this experience?*

- *Probing questions (if needed):*
  - *Can you tell me a little more about that?*

1. *Is there anything else you would like to add about your experience that I haven’t asked about?*

**WRAP UP**

**Aim:** To make consumers feel valued and give them clarity on what will happen next

- *Thank you so much for speaking with me today, Free will be in touch with further information about the next steps for this project.*
- *Would you like to receive a written copy of this interview to check that it reflects your experiences the way you intended?*
- *Would you like to see the de-identified analysis of all of the interviews so that you can make sure that it resonates with your experiences?*
- Thank them and sign off

## References

1. Australian Clinical Trials Alliance. Consumer involvement and engagement toolkit. Consumer involvement and engagement toolkit. Published 2020. Accessed May 20, 2020. https://involvementtoolkit.clinicaltrialsalliance.org.au/

2. Bossen C, Dindler C, Iversen O. *User Gains and PD Aims: Assessment from a Participatory Design Project*.; 2010:150. doi:10.1145/1900441.1900461

3. Bowen S, McSeveny K, Lockley E, Wolstenholme D, Cobb M, Dearden A. How was it for you? Experiences of participatory design in the UK health service. *CoDesign*. 2013;9(4):230-246. doi:10.1080/15710882.2013.846384

4. Haines K, Holdsworth C, Cranwell K, et al. Development of a Peer Support Model Using Experience-Based Co-Design to Improve Critical Care Recovery. *Crit Care Explor*. 2019;1(3). doi:10.1097/CCE.0000000000000006

5. Leask CF, Sandlund M, Skelton DA, et al. Framework, principles and recommendations for utilising participatory methodologies in the co-creation and evaluation of public health interventions. *Res Involv Engagem*. 2019;5(1):2. doi:10.1186/s40900-018-0136-9

6. Man M, Abrams T, McLeod R. *Implementing and Evaluating Co-Design: A Step-by-Step Toolkit*. NPC; 2019:20.

7. Pallesen KS, Rogers L, Anjara S, Brún AD, McAuliffe E. A qualitative evaluation of participants’ experiences of using co-design to develop a collective leadership educational intervention for health-care teams. *Health Expect*. 2020;23(2):358-367. doi:https://doi.org/10.1111/hex.13002
