# Supplementary material for: Development and Evaluation of a Framework for Authentic Online Co‐Design: Partnership‐Focussed Principles‐Driven Online Co‐Design
Source: Health Expect. 2024 Jul 9;27(4):e14138. doi: 10.1111/hex.14138 (PMC11233779; doi:10.1111/hex.14138)
Supplement: Supplementary file 4 — Supporting information. [file HEX-27-e14138-s005.docx]

# Appendix D: Details of qualitative data analysis

Although having more than one analyst is not considered necessary in reflexive thematic analysis, due to the deep engagement of F.C. in all aspects of the co-design and evaluation, a collaborative approach was taken to ensure that the analysis remained open to different perspectives and to enhance reflexive processes^1^. All three analysts have experience in qualitative research methods, with C.M. and K.C. having specific experience in Braun and Clarke's reflexive thematic analysis^1^.

Step 1: Dataset familiarisation.

All analysts familiarised themselves with the entire dataset by listening to the audio recordings and/or reading the transcripts, and made notes of initial insights that arose during this stage (both throughout the data and of the dataset as a whole).

Step 2: Data coding.

An inductive approach was used to facilitate an empathic, experiential approach to the analysis. Interesting or meaningful segments of data that appeared to answer the research question, were labelled with one or two words that captured a key concept or idea. F.C. coded all interviews, while C.M. and K.C. coded two interviews each. Qualitative data analysis software NVivo v12^2^ was used to organise and manage the data through the coding phase.

Step 3: Initial theme generation.

All three analysts met to review the codes generated in Step 2. Collaboratively, they grouped codes that appeared to share a core concept and illuminated an aspect of the research phenomenon. This resulted in five candidate themes, with associated coded data.

Step 4: Theme development and review.

F.C. synthesised the associated data for each theme and prepared a draft for review. The analysts met a second time to assess the fit of the candidate themes to the overall analysis by checking the relevance of the candidate themes to both the associated coded extracts and the full dataset. They also considered the scope of each theme and clearly articulated what would be included, and not included under each, resulting in four final themes.

Step 5: Theme refining, defining and naming.

F.C. then prepared another draft, and all three analysts met a third time to review and further refine the themes, their descriptions and finalise theme names.

Step 6: Writing up.

F.C. prepared the draft manuscript with all authors providing feedback and revisions.

##### Reflexive Statement

This statement aims to inform the reader as to the actions taken to sustain a reflective practice and describe influences on the interpretation of the qualitative data. Reflexivity was sustained throughout the design and conduct of this research by reflecting during fortnightly meetings with the core research team (F.C., A.S., R.T. and K.S), and via meetings and email contact between the co-analysts (F.C., C.M. and K.C) during the qualitative analysis and write up stage.

All three analysts are physiotherapists with backgrounds in coaching recreational physical activity for children. They place significant value on inclusive opportunities for physical activity, with both F.C. and K.C. having undertaken Doctor of Philosophy degrees on this topic. Furthermore, F.C.'s two-decade experience teaching circus activities meant they had preconceived notions of "what works” and worked consciously through the research co-design workshops to not utilise their experience as a coach to influence the design, and to prioritise the perspectives of the other circus coaches present. C.M. and K.C. also have prior experience in research co-design. Along with all three analysts' passion for genuine inclusion in co-design, during the analysis, attention was paid to negative cases and suggestions for improvements to P-POD were incorporated into the thematic findings.

## Project lead's detailed reflexive statement

The following reflexive statement from the project lead and primary analyst (F.C.) presents an overview of the values and assumptions that F.C. bought to this research and the interpretation of the findings and is thus written in first person.

This research sits in a constructivist ontology and epistemology. As both a feminist and a physiotherapist, allowing space for diverse stakeholder voices, and particularly those traditionally not given power in health research and decision-making, was extremely important to me throughout this research. I identified with three of the four different stakeholder groups in the co-design: I am a circus coach, a clinician, and a researcher, and although I am a mother, I have never parented a child born preterm. Creating a space where these parents could feel comfortable and valued and important was so fundamentally essential to me. However, having strong values of truth-seeking and desire for clarity (both personally and in my clinical work), meant I needed to consistently reflect on whether I was continuing to be open to multiple realities and resisting a positivist, objective lens. Certainly, over my qualitative journey as a researcher I have become much more comfortable sitting in the discomfort of remaining open to change throughout the analytic process in order to produce a strong, complex and thoughtful interpretation. Becoming comfortable with discomfort is a key learning I took into the co-design process and continued to learn from in this space. Slowing down, and valuing the process and relationships as much as the outcome was another key lesson I took away from the co-design, and continue to implement in my personal and professional life.

My assumption going into the co-design process was that we wouldn't be able to please everyone with the end design, and that people would be unhappy if they needed to compromise. I think my biggest learning in this space is that with transparent and truly collaborative decision-making processes, and people's respect for each other’s expertise (lived or professional), that the end design is not a product of compromises, but rather a product of each person's knowledge, creativity, and adaptability. This has changed my career path as a researcher, as I now actively seek out collaborative work as I value it so highly.

The experience that I was unprepared for in P-POD, which also shapes my future practice, was the extent of the emotional labour required to project lead and facilitate this process. I felt a huge responsibility to the team members to make sure that each participant felt included and involved in a way that made them feel capable, satisfied, and cared for. I also felt a huge responsibility to the children and families that would be the end-users of this program. This sense of responsibility meant that I bought absolutely everything I had to this project, both in the planning and implementation and the work between sessions, leaving very little emotional energy for other aspects of my personal and professional life over that time. I think it's important to plan for this emotional labour when considering engaging in co-design and include additional plans for your self-care as project lead and/or facilitator to ensure the sustainability of your emotional well-being throughout the process.

My assumptions going into the evaluation data analysis were that the co-design team members were going to be very critical of the process, and to have found it overwhelming and exhausting. As I had quite literally spent six months of my life trying to design a process that would be authentic and inclusive, I felt very sensitive to criticism and feelings of failure, so it took me a number of months to be brave enough to engage in the evaluation analysis due to my fear of negative feedback. Once I engaged with the data however, I quickly realised that this was not the primary experience of the team, and in fact, in listening to my own interview data, I was the only participant that strongly articulated these feelings. For this reason, choosing a collaborative approach to the analysis was essential to keep a sense of openness to the data, and to be able to work through any constructive criticism in a sensitive and future-focussed way.

## References

1. Braun V, Clarke V. *Thematic Analysis: A Practical Guide.* SAGE Publications; 2022. Accessed November 14, 2022. https://us.sagepub.com/en-us/nam/thematic-analysis/book248481

2. NVivo. Published online 2018. https://www.qsrinternational.com/nvivo-qualitative-data-analysis-software/home
