# Supplementary material for: Development and Evaluation of a Framework for Authentic Online Co‐Design: Partnership‐Focussed Principles‐Driven Online Co‐Design
Source: Health Expect. 2024 Jul 9;27(4):e14138. doi: 10.1111/hex.14138 (PMC11233779; doi:10.1111/hex.14138)
Supplement: Supplementary file 5 — Supporting information. [file HEX-27-e14138-s006.docx]

# Appendix E: Resulting co-designed intervention presented according to the TIDieR Checklist (adapted from Hoffman et al., 2014)

| TIDieR criteria Item 1 Brief name: Provide the name or a phrase that describes the intervention. |
| --- |
| CirqAll: preschool circus for premmies.   - Giving children born preterm extra opportunities to confidently work on their developmental goals through a fun interactive class, in a safe, fun environment. - Specialised circus program designed for the physical and mental well-being of pre-school children who were born preterm. - A place for children born preterm to be the best that they can be. - A place for families and kids to work on physical strength and confidence in a fun way.   This intervention has 3 components:   1. Training for program providers (coaches) 2. Introductory sessions for children born preterm and their families 3. Integrated continuous circus classes with term-born peers |

| TIDieR criteria Item 2 Why: Describe any rationale, theory, or goal of elements essential to the intervention. |
| --- |
| Preschool-aged children (three to five years old) born preterm (<37 weeks’ gestation) participate in less physical activity and less community-based activities than their term-born peers. Increasing participation in recreational physical activity may result in improved motor outcomes and favourable cardiometabolic and psychosocial outcomes. Circus training is non-competitive, non-gendered and can be practiced all year round. Circus training incorporates fundamental motor and social skills and embraces a creative approach to skill development rather than emphasizing a ‘right or wrong’ way of doing things. Circus training shows positive effects in at-risk school-aged and adult populations, some of whom are living with disability. Families, health professionals, circus coaches and academics co-designing a circus program for children born preterm may improve intervention uptake, knowledge translation and enhanced patient outcomes. Co-designed community-based circus activities may improve physical activity and community activity participation for children born preterm. Research with key stakeholders suggest that important elements to consider for this intervention are: professional development for the coaches delivering the program, and increased understanding of the preterm experiences; class structure modifications to enhance participation; and a developmentally therapeutic agenda.  **1. Training for program providers (coaches)** Goal: to increase coaches' confidence; to improve participation and outcomes of children and families.  **2. Introductory sessions for preterm children and their families** Goal of introductory sessions for preterm children only:   - allow the coach, child, and family to start to build a trusting relationship - allow the coach to get to know the child and their needs - allow the coach and family to discuss strategies that will enhance their participation in a circus class - enable the parent to discuss any concerns - have the child participate in a smaller version (30min) of the class to learn expectations, boundaries and key activities offered within the integrated program - provide an opportunity for parents to invite their child’s health worker to attend - child experiencing the environment beforehand   **3. Integrated continuous circus classes with term-born peers**  Goals:   - To welcome & support diversity within an integrated setting - Provide an environment similar to kinder or school settings - Focus on physical, social & behavioural developmental outcomes for the preschool age   This intervention should focus on: development & consolidation of key physical & social skills, building strength, trying new skills & activities, confidence building and perseverance.  These short-term goals will ideally result in the longer-term goals of: increased confidence and ability to participate in a broader range of physical activity, to socialise more easily, and to build resilience and confidence in everyday life.  It is important that this program: is fun & engaging with a focus on progressive challenges, meets kids where they are, has opportunities for parents to connect, has a welcoming environment that is about inclusion & acceptance. |

| TIDieR criteria Item 3 What (materials): Describe any physical or informational materials used in the intervention, including those provided to participants or used in intervention delivery or in training of intervention providers. |
| --- |
| Coach training topics (information provided during training of intervention providers):   - Understanding the preterm experience:   - Background of premature birth   - Barriers to participation   - Recognition of common impacts of prematurity   - Importance of intervention for this cohort   - Safety (manual handling, modifications, precautions, contraindications)   - Working with children with restricted mobility - Optimising a therapeutic agenda   - Developmental stages & milestones for pre-school aged children   - Motor learning/ planning theory   - Research informed goals/ goal setting and how to practically achieve them, including task analysis   - Class planning/ management   - Activity/task ideas for pre-schoolers born preterm - Enhancing meaningful inclusion   - Disability, diversity, and inclusion best practice   - Supporting alternative communication   - Supporting behaviour   - Supporting sensory processing   - Communication tactics for coaches engaging with parents/ support workers/ therapists   - Strategies for engaging & building confidence in pre-schoolers - CirqAll: Preschool circus for premmies   - Why circus?   - Why CirqAll?   - Guiding principles   Training is provided via an online platform, supported by interactive Zoom workshops. |

| TIDieR criteria Item 4 What (procedures): Describe each of the procedures, activities, and/or processes used in the intervention, including any enabling or support activities. |
| --- |
| 1. **Training for program providers (coaches)**    1. Radical flipped classroom approach: asynchronous learning (online) followed by synchronous application sessions (Zoom)    2. Topics as above 2. **Introductory sessions for preterm children and their families**    1. Component 2 consists of two introductory classes for preterm children and their parent/guardians offered during school holidays. The two classes are 60 minutes in length, each made up of a 30 minute ‘mini-class’ and 30 minutes of ‘getting to know you’ time with parents and children. The 'mini-class' is designed as part of Component 1 (in the online coach training) and will consist of familiarising children with key activities included in Component 3. The 'getting to know you' time, will involve supervised play while the parent and coach discuss ways to support the child, for optimal participation during Component 3. 3. **Integrated continuous circus classes with term-born peers**    1. Component 3 is a weekly 40-50min circus class in a mainstream, continuous term-based program. In component 3, children attend these classes alongside their term-born peers. As part of Component 1, coaches learn about key strategies for promoting inclusion in mainstream classes for children born preterm, and in Component 2, they have learnt from the parent and child what individual supports may be needed to optimise participation. |

| TIDieR criteria Item 5 Who provided: For each category of intervention provider (for example, psychologist, nursing assistant), describe their expertise, background and any specific training given. |
| --- |
| **Coach Training:** For: Experienced circus coaches  Providers: circus coaches, health professionals and parents  **Circus Program:** For: Preschoolers (3-5yos) born <37 weeks’ gestation. Providers: Experienced circus coaches who have completed the additional training, parent/ carer assistance when needed (tapered down over time) |

| TIDieR criteria  Item 6 How: Describe the modes of delivery (such as face to face or by some other mechanism, such as internet or telephone) of the intervention and whether it was provided individually or in a group.  Item 7 Where: Describe the type(s) of location(s) where the intervention occurred, including any necessary infrastructure or relevant features. |
| --- |
| **Coach Training: individual** asynchronous learning (online) followed by small group-based synchronous application sessions (Zoom)  **Circus Program:** Face-to-face small groups at community-based recreational circus schools |

| TIDieR criteria Item 8 When and how much: Describe the number of times the intervention was delivered and over what period of time including the number of sessions, their schedule, and their duration, intensity or dose. |
| --- |
| 1. **Training for program providers (coaches)**    1. TBC once content is written, important to offer flexibility with the synchronous Zoom sessions 2. **Introductory sessions for preterm children and their families**    1. Two 60min sessions over a 2-3 week period 3. **Integrated continuous circus classes with term-born peers**    1. A weekly rolling/ continuous program based on progressive challenge, 40-50min class duration |

| TIDieR criteria Item 9 Tailoring: If the intervention was planned to be personalised, titrated or adapted, then describe what, why, when, and how. |
| --- |
| Individual attention where needed, assistance provided by parent or another coach. Class content planned around each child's individual goals. |
