# Supplementary material for: Development and Evaluation of a Framework for Authentic Online Co‐Design: Partnership‐Focussed Principles‐Driven Online Co‐Design
Source: Health Expect. 2024 Jul 9;27(4):e14138. doi: 10.1111/hex.14138 (PMC11233779; doi:10.1111/hex.14138)
Supplement: Supplementary file 6 — Supporting information. [file HEX-27-e14138-s002.docx]

Appendix F: Final P-POD framework.


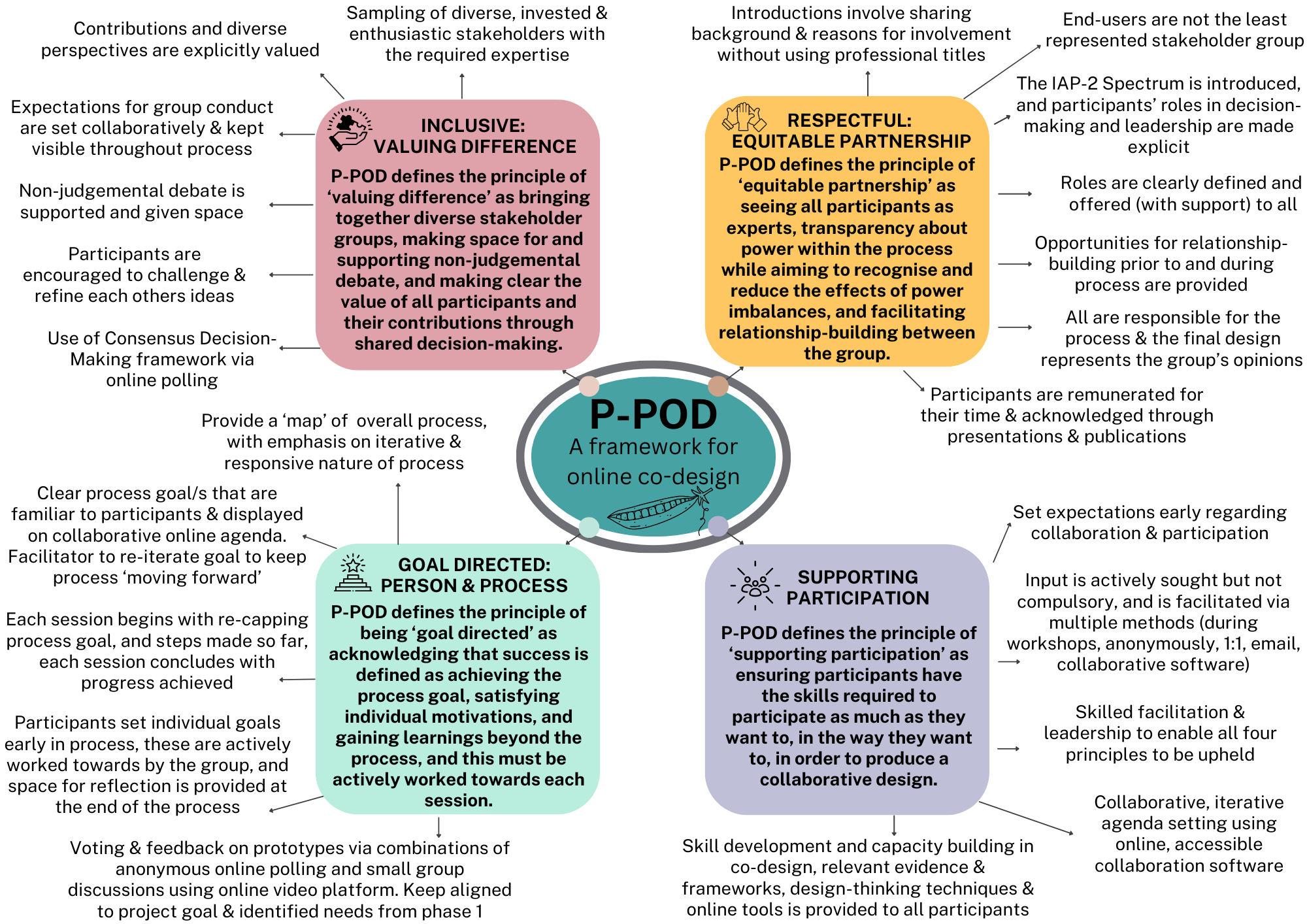


*Figure F1: The guiding principles and definitions of the final P-POD framework.*

*Table F1: Detailed final P-POD framework with associated strategies.*

| **P-POD principle & definition** | | **Example strategies across the co-design process** | **Where is this demonstrated?** |
| --- | --- | --- | --- |
| **Equitable partnership** | All participants are seen as experts in their lived and/ or professional experience. | Start each online meeting with Acknowledgement of Country and an acknowledgement of the appreciation and respect for the wealth of knowledge, experience, and skills in the ‘room’. | Meeting agendas (kept on openly available collaborative software e.g., Padlet).  Modelled by facilitators. |
|  |  | Participants are explicitly acknowledged for their expertise. | Remunerated for time contributed which is clearly described in recruitment process.  Authorship & co-presentation opportunities are offered to all members. |
|  | Transparency about power within the process while aiming to recognise & reduce the effects of power imbalances | Selecting group members so that there is higher number of end-users compared to other stakeholder groups. | Recruitment process. |
|  |  | During introductions, participants are asked not to include professional titles, but rather to introduce themselves with their relevant (to the current project) lived and/ or professional experience. | Explained in first meeting & modelled by facilitators. |
|  |  | Participants roles in decision-making within the co-design process is shared, transparent and explicitly described early *“we will partner in decision-making"* (IAP2 Spectrum – ‘collaborate’). Decision-Making processes use Consensus Decision-Making (CDM) via anonymous poll or REDCap survey & follow-up group discussion of results. | Decision-making process described early (e.g., Sessions 1-3). Copy of CDM framework image & link to further information uploaded to collaborative software. |
|  |  | Participants roles and choice in leadership are described clearly and early and revisited across the process. | IAP2 spectrum is introduced and P-POD positioned as a ‘collaborative’ process. Describe roles in the team, articulate the tasks involved, and ask for people to step forward. Emphasise they have choice about taking up these roles. This may take time, additional support and being revisited to be effective. |
|  |  | All are responsible for the process and the resulting design represents the groups' opinions – confirmed throughout process by checking prototypes with CDM and/ or annonymous polling. | Articulation of roles as above. Wrap-up by facilitator of key decisions/ activities that have been made in the session – asking for feedback if these have been mis-interpreted. Prototypes developed early and kept on collaborative software so all are aware of process and can re-direct in a timely way if needed. TIDieR checklist column is recommended for this. |
|  | Relationship building between the group is actively facilitated | Prior to co-design sessions:   - Consider sending each member a physical ‘welcome’ pack to create a sense of being part of a team. A notebook, pen, sticky note and some chocolate has worked well in the past. - Coming together to discuss thoughts and opinions about the project topic prior to the co-design process (particularly through an ‘information gathering phase’) can build a sense of familiarity with the facilitators, other group members, and the topic. | After recruitment, prior to/ aligned with early co-design sessions.  Incorporating an ‘information gathering phase’ with participants (e.g., qualitative study including a focus group).  Otherwise, consider structuring the first session with lots of small group sharing/ reflections from larger group work/ presentations. |
|  |  | During co-design sessions   - The start of each meeting includes a statement on the shared commitment of participants to the goal of the co-design process. - Breakout rooms for smaller group discussion are used often. - Ice-breaker activities are meaningful and useful. | Meeting agendas (kept on openly available collaborative software).  Modelled by facilitators.  Structuring of sessions. |
|  |  | After co-design sessions   - Holding a celebration event - Keeping team informed of futher steps | Space is made for celebration in whatever form suits the participants.  Regular updates (e.g., Yearly email) after the co-design concludes to update the team on what’s happening and offering further opportunities to be involved. |
| **Valuing difference** | Sampling of diverse, invested & enthusiastic participants with the required expertise | Clear articulation of project goal & relevance to participants is clear. | Advertising material.  Keep in first column of collaborative software so that visible to all group members and can be used as a guide for behaviour in the group. |
|  |  | Purposive sampling from different stakeholder groups (ideally from a prior 'Information Gathering Phase') | Study design & recruitment strategy. |
|  |  | May need to bring in additional participants as the scope of the intervention is developed. | Recruitment strategy. |
|  |  |  |  |
|  | Supports the involvement of all participants ; Participant’s contributions are heard and valued & there is space for non-judgemental debate | Ground rules set collaboratively using Padlet in the first session. Followed by a discussion on ‘how do we know if these things aren’t happening and how will we negotiate that?’ Rules are kept in the Padlet and viewed every meeting with time allowed to change/ amend if required. | Use as an ‘Ice Breaker’ activity as well as upskilling group in use of online collaborative software.  Keep in first column of Padlet so that visible to all group members and can be used as a guide for behaviour in the group. |
|  |  | A second facilitator positioned as a support person, both technical but also to clarify information/ activities or for feedback via private chat in Zoom or via phone call/ text. | Recruitment of support person/ second facilitator.  Explicit description of their role. |
|  |  | Responding to requests for further information/ clarification prior to subsequent sessions | Clear pathways of commuication described.  Timely responses to participant’s requests. |
|  |  | Diversity & inclusion is prioritised | Offer to include chosen pronouns as part of online name & modelled by academic stakeholders  Participants asked of accomodations required for participation and these are actioned. |
|  |  | Listening to and considering each idea presented, all ideas noted in Padlet | Strategies such as Nominal Group Technique, small group discussions, different people taking minutes, access to add to collaborative software at any time. |
|  |  | Participants are encouraged to refine & challenge ideas | Use of anonymous polling and up/down-voting ideas via collaborative software to evaluate prototypes  Design-thinking activities  Consensus Decision-Making (CDM) via anonymous Zoom poll or REDCap survey & follow-up group discussion of results. |
| **Supporting participation** | Ensure participants have the skills required to participate as much as they want to, in the way that they want to. | Participants upskilled in co-design processes, CDM framework & design thinking techniques during sessions.  Ensure all stakeholders have access to and are upskilled in each online platform/ software used | Short, impactful & relevant upskilling provided. The aim is a shared understanding & skill set.  Resources embedded into collaborative software, links for further reading/ learning provided.  Practice of techniques made explicit and graded in complexity. |
|  |  | Multiple methods of communication offered, e.g.,   - group-based Zoom workshops: verbal contributions, written in chat, anonymous in chat, private chat just to facilitators, on Padlet - outside of group Zoom workshops: part of anonymous feedback survey, emailed to facilitators, 1:1 session (Zoom or phone), on Padlet, via REDCap surveys | Clear verbal and written explanations of how participants can contribute.  Modelled by facilitators & built into agenda.  Reminders of options throughout process.  Ensuring Padlet accessibility to stakeholders at all times: Padlet was used as agenda and minutes tool, could be accessed by participants at any stage in the process (both during meetings and between). An offer was also made in the first session to send out agendas and minutes via email if preferred. |
|  | Participation is expected, meaningful and fun | Set expectations early regarding participation & collaboration. | Agenda item in first meeting.  Discussed openly throughout.  Shared roles in sessions; articulating goals and process clearly with stakeholder choice regarding roles |
|  |  | Encouraging input through various creative techniques | Utilise design thinking techniques via collaborative software such as idea storms, journey mapping, and add/delete/merge techniques |
|  |  | Open & responsive sessions | Anonymous REDCap quality improvement survey embedded into meetings; feedback enacted prior to subsequent sessions.  Collaborative agenda setting via collaborative software. |
| **Goal directed** | Clear objectives with attention to process and individual goals | Process goal is clear | The scope of the process is clearly outlined during the first meeting, the process goal is recorded in the collaborative software and re-articulated at the start of each meeting |
|  |  | Provide an overall ‘map’ of process with emphasis on responsive and iterative nature of process | Visualisation of co-design process provided on collaborative software & used to guide agenda-setting |
|  |  | Process stays in scope | Consider using ‘non-negotiables’ of the design as a way to ‘check in’ on the design at the mid-way point. |
|  |  | Individual motivations and goals are discussed | In the first session participants shared what they were hoping to get from the process and these individual goals were recorded in the collaborative software.  Become part of the ‘Celebration’ evant at the end of the process. |
|  | Each session moves the design forward in an iterative manner | Have a clear framework to guide design | Use of the TIDieR checklist^3^ to guide design, kept on Padlet^4^ and available to all participants at all times |
|  |  | Recap & reflect at the start and beginning of each workshop | Key decisions and latest prototypes recapped at the beginning of each session to direct thinking.  Each session finishes with member checking via reflection on the main findings of the session and how the session had moved the design forward. |
|  |  | Iterative process with ideas being refined and challenged | Use of anonymous polling (REDCap or Zoom) and up/down-voting of ideas via collaborative software to evaluate prototypes.  Small breakout rooms to create space for quieter voices. |
